# Why Women Leave Medicine: The Missing Life Skills We Never Teach

**DOI:** 10.12669/pjms.42.2.15392

**Published:** 2026-02

**Authors:** Shaukat Ali Jawaid, Mariyah Hidayat, Noor-i-Kiran Naeem

**Affiliations:** 1 Shaukat Ali Jawaid Chief Editor, Pakistan Journal of Medical Sciences, Karachi, Pakistan; 2 Prof. Dr. Mariyah Hidayat, MPhil., PhD. Professor of Anatomy, University College of Medicine and Dentistry, University of Lahore, Lahore, Pakistan; 3 Dr. Noor-i-Kiran Naeem MBBS, FCPS MSc. MEd (UOL), Ph.D.MEd. Associate Professor of Medical Education ABWA Medical College, Faisalabad, Pakistan

Every year, thousands of bright young women enter medical colleges in Pakistan, yet many do not remain in the workforce long enough to build careers. The journey of a woman doctor does not end at her graduation, as for many, that is where the struggle truly begins in reality. The undergraduate studies of Medicine and Surgery teaches dozens of clinical competencies, yet nothing prepares young women for what follows marriage: childcare, family expectations, household responsibilities, and the domestic realities that determine whether women can continue in the profession after marriage. These are not innate abilities; they are life skills that require training, guidance, and shared responsibility. Yet our schools, families, training institutions, and society in general, seldom equip young women and equally, young men, with these competencies. The gap between academic achievement and real-world preparedness explains, in part, why the professional dropout rate of women physicians remains high and why so few eventually rise to supervisory, administrative, or leadership roles in academic medicine.

The challenge is not ambition but alignment. Medicine demands consistancy, whereas families demand time and care. When systems do not allow both, women leave, not because they lack ability, but because the cost of staying becomes too high. This is how a nation loses its physicians, not in examinations or interviews, but at the kitchen table. The result is a persistent gender gap in the medical workforce. Although women dominate medical school admissions, a fraction ascend to become consultants, fellows, researchers, or academic leaders. This is not a failure of talent or ambition; it is a failure to equip families and institutions with the life skills and systems that make careers possible after marriage.

Yet awareness alone does not drive change. Conversations on this subject have existed for decades, but largely within private domains, which includes family gatherings, informal groups, and whispered concerns in hospital corridors. What was missing was an academic vocabulary to describe the problem and a public forum to acknowledge its consequences for the medical workforce. The first attempt to give language to this topic surfaced during a Certificate in Medical Editing (CME) assignment at the University of Health Sciences, Lahore, conducted annually in collaboration with the Pakistan Association of Medical Editors (PAME).^1^ The first author proposed the subject to participants of CME Batch-4 in 2022 consisting predominantly of women physicians, as a topic for their special assignment. Surprisingly, not a single participant selected it. Their hesitation was revealing. The topic was relevant, but also personal and complicated, and not easy to put into words. Eventually, he wrote an editorial on the subject and shared it with six senior female professors from different institutions to seek their feedback prior to publication.^2^ Several valuable suggestions emerged, as they offered constructive and pragmatic solutions, which included flexible training structures, campus daycare facilities, and institutional mechanisms that acknowledged the realities of parenting. Not long after, another scholarly piece by Prof. Aisha Zaheer shifted the conversation further by highlighting why a majority of female medical graduates leave the profession.^3^ Momentum followed and the Medical Women’s Association of Pakistan (MWAP), under the dynamic leadership of Dr. Wajiha Rizwan and her colleagues, transformed the discussion into organized advocacy. They highlighted professional barriers faced by women physicians and introduced the term *Gender Professionalism,^4^* a lens through which expectations, respect, and role negotiation in healthcare could be examined. They also addressed the issue of the so-called “Doctor Bride” and worked to dispel this misconception.^5^,^6^,^7^

An editorial by Medical Women’s Association of Pakistan (MWAP) also addressed the issue of “Bride Doctors” exploring the barriers to women’s participation in medicine, the challenges of retention, and obstacles to thriving the leadership roles.^8^

Furthermore, they initiated various programs for professional capacity building and highlighted the achievements of Pakistani women physicians at national and international forums. Gradually, what was once a whispered concern became an acknowledged workforce issue. Qualitative research and public discussions have since added depth, revealing that female physicians must navigate domestic expectations at home and institutional hostility in the workplace. Recent studies show that women physicians face not only domestic pressures but also workplace harassment, violence, and limited institutional support, indicating systemic rather than individual barriers.^9^,^10^

Life skills cannot be directed toward young women alone. Parenting, household management, and the emotional labor of caregiving are shared responsibilities, yet in Pakistani families these burdens fall overwhelmingly on women. Boys and young men, despite being equal beneficiaries of stable homes, are seldom trained for partnership and caregiving. Without parallel preparation, marriage exposes unequal life skills, and women’s careers absorb the cost through reduced participation, delayed training, or early exit from the workforce.

The evolving role of women in healthcare makes this conversation urgent. Female physicians are expected to manage patient care, examinations, and research while also fulfilling expectations of childcare, social obligations, and extended family involvement. Yet medical curricula do not prepare them, or their spouses, for negotiation, communication, conflict resolution, emotional regulation, time management, or boundary setting. These competencies influence workforce retention more than clinical competence alone.

Academic progression demands more than clinical competence. It requires research, publications, and ongoing professional development. Social media can help with visibility, but it does not carry the weight of scholarship. Many younger physicians discover this too late and find academic pathways difficult to enter. The capacity to plan ahead, manage time, and stay focused is not innate. These learned life skills determine who remains in the academic track and who quietly steps away. Yet medical education offers little guidance on such competencies. Institutions cannot remain indifferent. Flexible scheduling models, on-site childcare, exemptions from night duties when necessary, and structured remuneration options retain women in the workforce and enable them to pursue specialist training without eroding family life. Policy reforms are important, but family support is equally decisive. Institutions may open doors, but it is the home that decides whether a woman walks through them. In the end, a spouse can make or break a career.

Ultimately, retaining women in medicine is not a “woman’s issue.” It is a systems issue, a family issue, and increasingly, a national workforce sustainability issue. Pakistan invests heavily in training medical students; losing half its graduates to preventable attrition is a luxury the country cannot afford. Training boys and girls timely in life skills offers one of the most practical pathways toward shared parenting, healthier marriages, and stable professional careers. If families, institutions, medical curricula, and policy frameworks evolve together, the question will no longer be whether women can remain in medicine after marriage, but why we waited so long to make it possible.
